# 5-(4-Chloro­benz­yl)-1*H*-tetra­zole

**DOI:** 10.1107/S1600536811028388

**Published:** 2011-07-23

**Authors:** Pei-Jiang Liu, Dong-Sheng Ma, Shuai Zhang, Guang-Feng Hou

**Affiliations:** a College of Chemistry and Materials Science, Heilongjiang University, Harbin 150080, People’s Republic of China

## Abstract

In the title compound, C_8_H_7_ClN_4_, the phenyl and tetra­zole rings are inclined at a dihedral angle of 67.52 (6)°. In the crystal, mol­ecules are linked by an N—H⋯N hydrogen bond into a chain structure along [010]. π–π inter­actions with centroid–centroid distances of 3.526 (1) Å between adjacent tetra­zole rings further link the chains, forming a ribbon structure.

## Related literature

For background to tetra­zole compounds, see: Kitagawa *et al.* (2004 ▶); Zhao *et al.* (2008 ▶); For the synthesis, see: Luo *et al.* (2006 ▶).
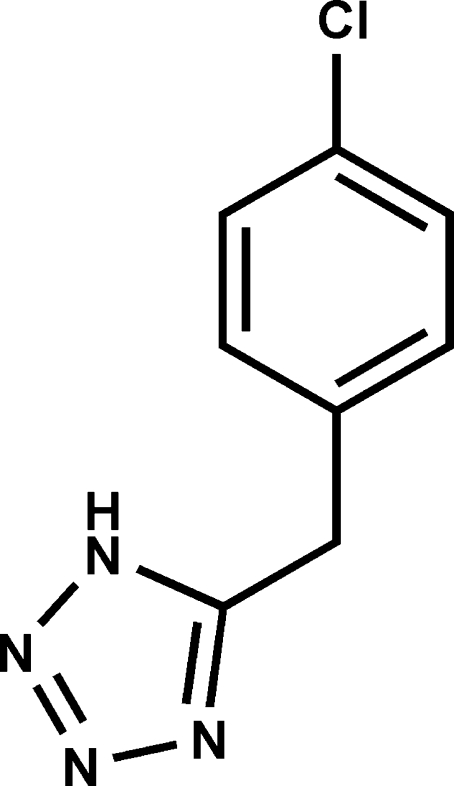


## Experimental

### 

#### Crystal data

C_8_H_7_ClN_4_
*M* *_r_* = 194.63
Monoclinic, 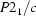

*a* = 14.654 (3) Å
*b* = 4.9321 (10) Å
*c* = 12.688 (3) Å
β = 105.63 (3)°
*V* = 883.1 (3) Å^3^
*Z* = 4
Mo *K*α radiation
μ = 0.39 mm^−1^
*T* = 293 K
0.40 × 0.38 × 0.15 mm

#### Data collection

Rigaku R-AXIS RAPID diffractometer
Absorption correction: multi-scan (*ABSCOR*; Higashi, 1995 ▶) *T*_min_ = 0.860, *T*_max_ = 0.944
8039 measured reflections
2015 independent reflections
1546 reflections with *I* > 2σ(*I*)
*R*_int_ = 0.025

#### Refinement

*R*[*F* ^2^ > 2σ(*F* ^2^)] = 0.033
*wR*(*F* ^2^) = 0.094
*S* = 1.08
2015 reflections
122 parameters
1 restraint
H atoms treated by a mixture of independent and constrained refinement
Δρ_max_ = 0.18 e Å^−3^
Δρ_min_ = −0.33 e Å^−3^

### 

Data collection: *RAPID-AUTO* (Rigaku, 1998 ▶); cell refinement: *RAPID-AUTO*; data reduction: *CrystalStructure* (Rigaku/MSC, 2002) ▶; program(s) used to solve structure: *SHELXS97* (Sheldrick, 2008 ▶); program(s) used to refine structure: *SHELXL97* (Sheldrick, 2008 ▶); molecular graphics: *SHELXTL* (Sheldrick, 2008 ▶); software used to prepare material for publication: *SHELXL97*.

## Supplementary Material

Crystal structure: contains datablock(s) I, global. DOI: 10.1107/S1600536811028388/ng5199sup1.cif

Structure factors: contains datablock(s) I. DOI: 10.1107/S1600536811028388/ng5199Isup2.hkl

Supplementary material file. DOI: 10.1107/S1600536811028388/ng5199Isup3.cml

Additional supplementary materials:  crystallographic information; 3D view; checkCIF report

## Figures and Tables

**Table 1:** Hydrogen-bond geometry (Å, °)

*D*—H⋯*A*	*D*—H	H⋯*A*	*D*⋯*A*	*D*—H⋯*A*
N4—H1⋯N1^i^	0.90 (1)	1.92 (1)	2.8013 (15)	168 (2)

Symmetry code: (i) 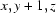.

**Fig. 1.**
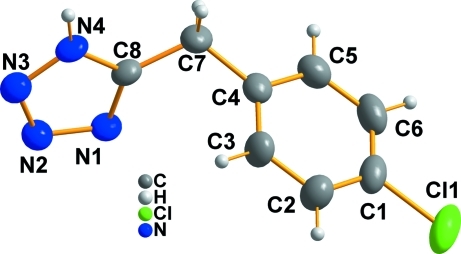
The molecular structure of the title compound, showing displacement ellipsoids at the 50% probability level for non-H atoms.

**Fig. 2.**
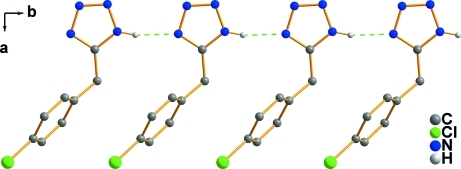
A partial packing view, showing chain structure along [0 1 0].

**Fig. 3.**
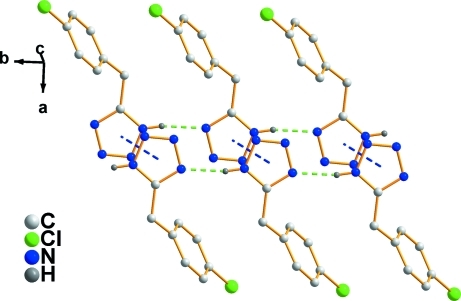
A partial packing view, showing double chain structure forming by N—H···N hydrogen bonds and π—π intercations.

## supplementary crystallographic information

### Comment

The tetrazole has attracted considerable interesting owing to their structural characterization in coordination chemistry and the extensively application in medicinal chemistry and materials science (Zhao *et al.* 2008; Kitagawa *et al.* 2004). Here, we report the synthesis and crystal structure of the title compound.

As shown in fig.1, the benzenyl plane and tetrazole rings form a dihedral angle about 67.52 (6) ° (Fig. 1). In the crystal packing, the molecules are linked by N—H···N hydrogen bonds into a chain structure alone [010] (Fig. 2, Table 1). The π—π interactions with distances of 3.526 (1) Å (center to center) between the adjacent tetrazole rings further link them to form ribbon structure (Fig. 3).

### Experimental

The title compound was prepared as follows (Luo *et al.* 2006):2-(4-chlorophenyl)acetonitrile (6.06 g, 0.04 mol), NaN3 (3.9 g, 0.06 mol) and NH_4_Cl (3.21 g, 0.06 mol) were dissolved in DMF (120 ml). The mixture was reflux for 20 h under stirring. Then, it was cooled to room temperature and the mixture was filtered. The solvent was evaporated and the residue was poured into cold water (30 ml) to give the title compound (4.32 g, 55.5 %). The crystals suitable for X-ray diffraction were obtained from 10 mL mixed solution of ethanol and water (1:1).

### Refinement

The anormal reflection data (-12 3 3) have been omitted during the refinement.H atoms bound to C atoms were placed in calculated positions and treated as riding on their parent atoms, with C—H = 0.93 Å (aromatic); C—H = 0.97 Å (methylene), and with *U*_iso_(H) = 1.2*U*_eq_(C). N-bounded H atom was found from Fourier map and was refined restrainedly with N—H = 0.90 Å.

### Crystal data

**Table:**

C_8_H_7_ClN_4_	*F*(000) = 400
*M**_r_* = 194.63	*D*_x_ = 1.464 Mg m^−^^3^
Monoclinic, *P*2_1_/*c*	Mo *K*α radiation, λ = 0.71073 Å
Hall symbol: -P 2ybc	Cell parameters from 6142 reflections
*a* = 14.654 (3) Å	θ = 3.3–25.1°
*b* = 4.9321 (10) Å	µ = 0.39 mm^−^^1^
*c* = 12.688 (3) Å	*T* = 293 K
β = 105.63 (3)°	Block, colorless
*V* = 883.1 (3) Å^3^	0.40 × 0.38 × 0.15 mm
*Z* = 4	

### Data collection

**Table:**

Rigaku R-AXIS RAPID diffractometer	2015 independent reflections
Radiation source: fine-focus sealed tube	1546 reflections with *I* > 2σ(*I*)
graphite	*R*_int_ = 0.025
ω scans	θ_max_ = 27.5°, θ_min_ = 3.3°
Absorption correction: multi-scan (*ABSCOR*; Higashi, 1995)	*h* = −18→19
*T*_min_ = 0.860, *T*_max_ = 0.944	*k* = −6→6
8039 measured reflections	*l* = −16→16

### Refinement

**Table:**

Refinement on *F*^2^	Primary atom site location: structure-invariant direct methods
Least-squares matrix: full	Secondary atom site location: difference Fourier map
*R*[*F*^2^ > 2σ(*F*^2^)] = 0.033	Hydrogen site location: inferred from neighbouring sites
*wR*(*F*^2^) = 0.094	H atoms treated by a mixture of independent and constrained refinement
*S* = 1.08	*w* = 1/[σ^2^(*F*_o_^2^) + (0.0482*P*)^2^ + 0.0994*P*] where *P* = (*F*_o_^2^ + 2*F*_c_^2^)/3
2015 reflections	(Δ/σ)_max_ = 0.001
122 parameters	Δρ_max_ = 0.18 e Å^−^^3^
1 restraint	Δρ_min_ = −0.33 e Å^−^^3^

### Special details

**Table:**

Geometry. All esds (except the esd in the dihedral angle between two l.s. planes) are estimated using the full covariance matrix. The cell esds are taken into account individually in the estimation of esds in distances, angles and torsion angles; correlations between esds in cell parameters are only used when they are defined by crystal symmetry. An approximate (isotropic) treatment of cell esds is used for estimating esds involving l.s. planes.
Refinement. Refinement of F^2^ against ALL reflections. The weighted R-factor wR and goodness of fit S are based on F^2^, conventional R-factors R are based on F, with F set to zero for negative F^2^. The threshold expression of F^2^ > 2sigma(F^2^) is used only for calculating R-factors(gt) etc. and is not relevant to the choice of reflections for refinement. R-factors based on F^2^ are statistically about twice as large as those based on F, and R- factors based on ALL data will be even larger.

### Fractional atomic coordinates and isotropic or equivalent isotropic displacement parameters (Å^2^)

**Table:**

	*x*	*y*	*z*	*U*_iso_*/*U*_eq_	
C1	0.37038 (9)	0.2990 (3)	1.07319 (14)	0.0463 (4)	
C2	0.29853 (11)	0.4022 (3)	1.11205 (14)	0.0503 (4)	
H2	0.2911	0.3450	1.1791	0.060*	
C3	0.23731 (10)	0.5923 (3)	1.05032 (13)	0.0465 (4)	
H3	0.1891	0.6638	1.0768	0.056*	
C4	0.24675 (9)	0.6773 (3)	0.95005 (12)	0.0380 (3)	
C5	0.31899 (11)	0.5667 (3)	0.91227 (15)	0.0467 (4)	
H5	0.3259	0.6203	0.8446	0.056*	
C6	0.38090 (11)	0.3781 (3)	0.97347 (15)	0.0518 (4)	
H6	0.4292	0.3057	0.9473	0.062*	
C7	0.18309 (10)	0.8938 (3)	0.88444 (14)	0.0451 (4)	
H7A	0.2059	1.0694	0.9149	0.054*	
H7B	0.1887	0.8888	0.8100	0.054*	
C8	0.08089 (9)	0.8704 (2)	0.88086 (11)	0.0311 (3)	
Cl1	0.44739 (3)	0.06175 (9)	1.15206 (5)	0.0707 (2)	
N1	0.03034 (8)	0.6471 (2)	0.87394 (9)	0.0347 (3)	
N2	−0.06056 (8)	0.7269 (2)	0.86416 (10)	0.0393 (3)	
N3	−0.06566 (8)	0.9885 (2)	0.86545 (10)	0.0404 (3)	
N4	0.02296 (8)	1.0797 (2)	0.87674 (9)	0.0346 (3)	
H1	0.0343 (11)	1.2592 (6)	0.8810 (12)	0.048 (4)*	

### Atomic displacement parameters (Å^2^)

**Table:**

	*U*^11^	*U*^22^	*U*^33^	*U*^12^	*U*^13^	*U*^23^
C1	0.0327 (7)	0.0365 (7)	0.0624 (10)	0.0041 (6)	0.0001 (6)	−0.0060 (7)
C2	0.0506 (9)	0.0497 (9)	0.0504 (10)	0.0101 (7)	0.0130 (7)	0.0055 (7)
C3	0.0416 (8)	0.0477 (9)	0.0531 (10)	0.0127 (7)	0.0176 (7)	0.0025 (7)
C4	0.0330 (6)	0.0302 (7)	0.0494 (9)	−0.0047 (6)	0.0090 (6)	−0.0018 (6)
C5	0.0424 (8)	0.0469 (9)	0.0548 (10)	−0.0032 (7)	0.0198 (7)	−0.0019 (7)
C6	0.0354 (7)	0.0486 (9)	0.0741 (12)	0.0022 (7)	0.0194 (8)	−0.0124 (8)
C7	0.0401 (7)	0.0327 (7)	0.0615 (10)	−0.0044 (6)	0.0119 (7)	0.0091 (7)
C8	0.0390 (6)	0.0224 (6)	0.0306 (7)	0.0005 (5)	0.0068 (5)	0.0001 (5)
Cl1	0.0512 (3)	0.0538 (3)	0.0907 (4)	0.0194 (2)	−0.0094 (2)	−0.0025 (2)
N1	0.0390 (6)	0.0229 (5)	0.0424 (7)	−0.0012 (5)	0.0115 (5)	−0.0019 (4)
N2	0.0390 (6)	0.0329 (6)	0.0473 (7)	−0.0002 (5)	0.0140 (5)	−0.0006 (5)
N3	0.0429 (6)	0.0337 (6)	0.0465 (7)	0.0059 (5)	0.0153 (5)	0.0025 (5)
N4	0.0461 (6)	0.0209 (5)	0.0366 (7)	0.0024 (5)	0.0107 (5)	0.0003 (4)

### Geometric parameters (Å, °)

**Table:**

C1—C6	1.372 (2)	C6—H6	0.9300
C1—C2	1.375 (2)	C7—C8	1.4906 (19)
C1—Cl1	1.7423 (16)	C7—H7A	0.9700
C2—C3	1.385 (2)	C7—H7B	0.9700
C2—H2	0.9300	C8—N1	1.3169 (17)
C3—C4	1.381 (2)	C8—N4	1.3284 (17)
C3—H3	0.9300	N1—N2	1.3622 (16)
C4—C5	1.386 (2)	N2—N3	1.2927 (17)
C4—C7	1.5117 (19)	N3—N4	1.3449 (17)
C5—C6	1.383 (2)	N4—H1	0.8998 (11)
C5—H5	0.9300		
			
C6—C1—C2	120.83 (14)	C5—C6—H6	120.3
C6—C1—Cl1	120.30 (12)	C8—C7—C4	115.34 (12)
C2—C1—Cl1	118.87 (14)	C8—C7—H7A	108.4
C1—C2—C3	119.32 (16)	C4—C7—H7A	108.4
C1—C2—H2	120.3	C8—C7—H7B	108.4
C3—C2—H2	120.3	C4—C7—H7B	108.4
C4—C3—C2	121.06 (13)	H7A—C7—H7B	107.5
C4—C3—H3	119.5	N1—C8—N4	107.77 (11)
C2—C3—H3	119.5	N1—C8—C7	127.54 (12)
C3—C4—C5	118.32 (14)	N4—C8—C7	124.55 (12)
C3—C4—C7	121.43 (13)	C8—N1—N2	106.44 (10)
C5—C4—C7	120.20 (14)	N3—N2—N1	110.23 (11)
C6—C5—C4	121.15 (16)	N2—N3—N4	106.11 (11)
C6—C5—H5	119.4	C8—N4—N3	109.45 (11)
C4—C5—H5	119.4	C8—N4—H1	131.0 (11)
C1—C6—C5	119.31 (14)	N3—N4—H1	119.5 (10)
C1—C6—H6	120.3		

### Hydrogen-bond geometry (Å, °)

**Table:**

*D*—H···*A*	*D*—H	H···*A*	*D*···*A*	*D*—H···*A*
N4—H1···N1^i^	0.90 (1)	1.92 (1)	2.8013 (15)	168.(2)

Symmetry codes: (i) *x*, *y*+1, *z*.
